# How does emotional wellbeing relate to underachievement in a general population sample of young adolescents: a neurocognitive perspective

**DOI:** 10.3389/fpsyg.2013.00673

**Published:** 2013-09-27

**Authors:** Tamara van Batenburg-Eddes, Jelle Jolles

**Affiliations:** Department of Educational Neuroscience, Faculty of Psychology and Education, VU University AmsterdamAmsterdam, Netherlands

**Keywords:** emotional wellbeing, educational underachievement, adolescence, executive functioning, epidemiology

## Abstract

Underachievement in school during early adolescence predicts future economic and personal difficulties. Particular neurocognitive skills on the domain of executive functions start to mature during adolescence. This fact and the physical and psychological changes typical for the transition from childhood to adulthood make adolescents vulnerable to emotional problems. The current study investigated the relationship between mild emotional problems which are highly prevalent among adolescents and underachievement in school, and the role of neurocognitive functioning in this relation. This study was conducted in a substantial sample of typical developing young adolescents who just made the transition to secondary education. Pupils were on average 12.5 years old (standard deviation 0.5), and 45% of the included sample were girls. Emotional wellbeing was associated with underachievement [Odds ratio (OR) 5.15, 95% confidence interval (CI) 3.06–8.68] after adjusting for background variables. Self-reported neurocognitive functioning partly explained the relation between emotional wellbeing and underachievement (OR 2.22, 95% CI 1.23–3.99), yet, emotional wellbeing remained statistically associated with underachievement after correcting for additional confounders (OR 1.99, 95% CI 1.08–3.66). The observed findings suggest that emotional wellbeing plays an essential role in underachievement during the first year of secondary education.

## Introduction

Educational attainment is important from a societal and individual perspective. From a societal point of view, educated citizens are necessary to increase a nation's standard of living (Kessler et al., 1995). On the individual level, education accumulates knowledge, cognitive and motor skills and experiences, resources and habits. These shape personal control, a healthy lifestyle, income, employment, interpersonal relations and social support (Mirowsky and Ross, 2003). Moreover, education has an impact on subjective outcomes such as happiness and perceived quality of life (Hartog and Oosterbeek, 1998).

Underachievement is an important aspect of educational attainment, which is defined in many different ways (Smith, 2003). McCoach and Siegle (2003) claim that underachievement is most commonly defined as “a discrepancy between potential (or ability) and performance (or achievement)” (Dowdall and Colangelo, 1982). According to this definition, underachievers are at risk of failing to achieve their academic potential and of entering society with a lower educational level than possible, i.e., with unexploited capacities.

For this study, we focused on Dutch pupils who just made the transition from primary to secondary education. This sample is particularly interesting because in many countries this transition tends to be followed by a decrease in academic achievement. In addition, in the Netherlands the transition to secondary education takes place between the age of 11 and 13 which, in Dutch youth, approximately coincides with the average age at which puberty starts (Mul et al., 2001).

The onset of puberty is triggered by adolescent brain maturation processes with subsequent striking changes in body and behavior. Moreover, the changes that characterize adolescent brain development impact on higher cognitive functions, reasoning and interpersonal interactions, cognitive control of emotions, risk-vs.-reward appraisal and motivation (Paus et al., 2008), what often makes this period a turbulent time for adolescents, their parents and their social environment. Most adolescents find their way in becoming an independent and self-supporting member of society without too many difficulties. Yet, minor deviances in timing and magnitude from the typical changes in neural systems contribute to an increase in adolescents' vulnerability for feelings of hopelessness, worthlessness, and mental health problems (Kessler et al., 2005; Newman and Newman, 2008; Paus et al., 2008). The increased incidence of mental health problems during adolescence is probably partly explained by the interaction between deviances from typical adolescent maturation processes with psychosocial factors (for example school and relationships) and/or biological environmental factors, such as pubertal hormonal changes and drugs and abuse (Paus et al., 2008). Adolescence is considered to be a period of increased emotional instability. This emotional instability may predispose adolescents to withdraw themselves from participation and involvement in school, which can lead to lower grades, lower school attendance, and -even worse-, school dropout. In short, adolescents, due to the transition they are going through, are at risk to obtain a lower educational attainment. At the same time, adolescence is a period in which the educational base for the future is laid, as educational attainment is a well-established predictor of subjective and objective outcomes such as occupational status, income, physical and mental health, happiness and quality of life (Hartog and Oosterbeek, 1998; Mirowsky and Ross, 2003).

Therefore, we investigated in this study whether mild emotional problems are related to efforts and results in school, i.e., underachievement. The relation between emotional well-being and school performance has not been studied often, as most research focuses on the effects of mental health disorders on educational attainment (Kessler et al., 1995; Breslau et al., 2008; Lee et al., 2009). Within mental health disorders sometimes a distinction is made between externalizing and internalizing disorders. In general, externalizing disorders encompass behaviors that primarily are a problem for the environment; they are marked by defiance, impulsivity, disruptiveness, aggression, or over activity. In contrast, internalizing disorders are referred to as problems occurring in the person, and are characterized by withdrawal, anxiety or depressed mood (Hinshaw, 1992; Whitcomb and Merrell, 2013). Several studies suggest that externalizing disorders reduce educational attainment, whereas for internalizing disorders results are inconclusive. For example, in a large nationally representative study of the US adult population, Breslau et al. (2008) found that onset of externalizing disorders is most consistently related to premature termination of education. In a large-scale study that was part of the World Mental Health Survey Initiative in which 16 high-income and low-income countries participated, Lee et al. (2009) found similar results in the high-income countries. However, findings for anxiety disorders and mood disorders were less consistent but these disorders were specifically related to failing to graduate in high-school. In another study among 83 adolescents who met subthreshold criteria for depression, the effect of depressed mood on objective and subjective outcomes of school performance was investigated. This study found that depressive symptoms were not related to the objective outcomes but were related to perceptions of impairment in functioning, such as the ability to do well in school, concentrate on or complete their homework, to concentrate in class, attend class, and deal with other students (Humensky et al., 2010), i.e., the subjective outcomes.

As was described earlier, in typical adolescent maturation processes, changes in the brain play a substantial role (Giedd, 2008). Paus (2005) concludes in his review that regarding the morphometrical development of the adolescent brain, the overall volume of white matter increases steadily and linear throughout adolescence, whereas for gray matter the cortical volumes change in a non-linear fashion across different regions. Yet, little is known about how these changes in structure during adolescence relate to those in function. Prefrontal brain areas are one of the latest to mature, and these develop rapidly during adolescence. The prefrontal brain areas and large brain networks extending from prefrontal structures underlie the so-called “executive functions” (EF; Miller and Cohen, 2001). EF encompass cognitive processes that are essential for goal-oriented, efficient, and adaptive (social) behavior. Furthermore, EF are essential for an efficient adaptation to a changing environment. In addition, they enable a person to oversee short-term and long-term consequences of his or her behavior and to make decisions based upon proper evaluations of alternatives (Anderson et al., 2001; Lee et al., 2012). EF, such as inhibition processes, attention, self-control, planning and organizing (Hofmann et al., 2012), improve steadily throughout adolescence and well into adulthood with some skills reaching adult levels earlier than others. For example, more basic skills, such as attention, reach adult levels earlier (Anderson et al., 2001; Klenberg et al., 2001), whereas planning and problem-solving which are more complex skills show a protracted developmental trajectory (De Luca et al., 2003; Luna et al., 2004).

Research has shown deviances in the way the brain receives and processes information between adolescents with and without emotional problems (Zahn-Waxler et al., 2000). Emotional problems are in part characterized by problems with EF, for example, a lack of concentration or ruminating. In addition, EF are a seemingly obvious prerequisite for learning in school. Recent findings from developmental cognitive neuroscience suggest a strong influence of social context on the development of neural systems (Lenroot and Giedd, 2006; Crone and Dahl, 2012). Therefore, it is important to evaluate the extent to which EF play a role in the association between emotional wellbeing and school performance.

In this study, we included a substantial sample of typical developing adolescents in their first year of secondary school, who were thus, relatively homogeneous in age. We studied the association between emotional wellbeing and underachievement and, most importantly, the role of neurocognitive functioning in this relation. The substantial sample size enabled us to adjust for many confounders, such as demographic characteristics, cultural capital, and attitude toward teachers. The findings of this study have important implications for interventions aimed at creating a learning environment that enables adolescents to fully exploit their talents and to get the best out of their education.

We hypothesized that pupils who felt less well were more likely to underachieve in school, and we expected this association to be explained by self-reported neurocognitive functioning.

## Materials and methods

### Study population

This study was part of the LEERLIJN study, which is a large scale research project conducted in the Netherlands. The aim of the LEERLIJN study is to investigate skills, experiences, motivation and the social environment of young adolescents (mean age 12.5 years, SD 0.5) in their first year of secondary school. A substantial sample of pupils (*n* = 2215) was enrolled from 10 regular secondary schools; schools with varying educational levels, i.e., ranging from “pre-vocational secondary educational level” via “higher general educational level” to “pre-university secondary educational level.” In the current study, we were interested in the association between emotional wellbeing and the risk of underachievement. To study the risk of underachievement (i.e., the risk of failing to reach learning potential), we compared underachievers with high achieving pupils. Pupils were classified as an underachiever if: (1) both the pupil and the tutor agreed on a question about underperforming (“I think I can do much better in school than I do at the moment”/“This pupil can do much better than his or her rates currently show”), and (2) when their grades were low-to-average. High achieving pupils are pupils who performed, according to themselves or their tutor, in line with their capabilities, and who had high grades. A tutor is a teacher designated to coach a class; each class in secondary education in the Netherlands has a tutor. In addition to teaching, a tutor keeps track of individual pupils' progress, is in contact with other teachers and serves as a trusted counselor. The current study was employed in the second semester of the pupils first year of secondary education. Thus, pupils and tutors had 8 months to build their relationship.

**Figure 1:**
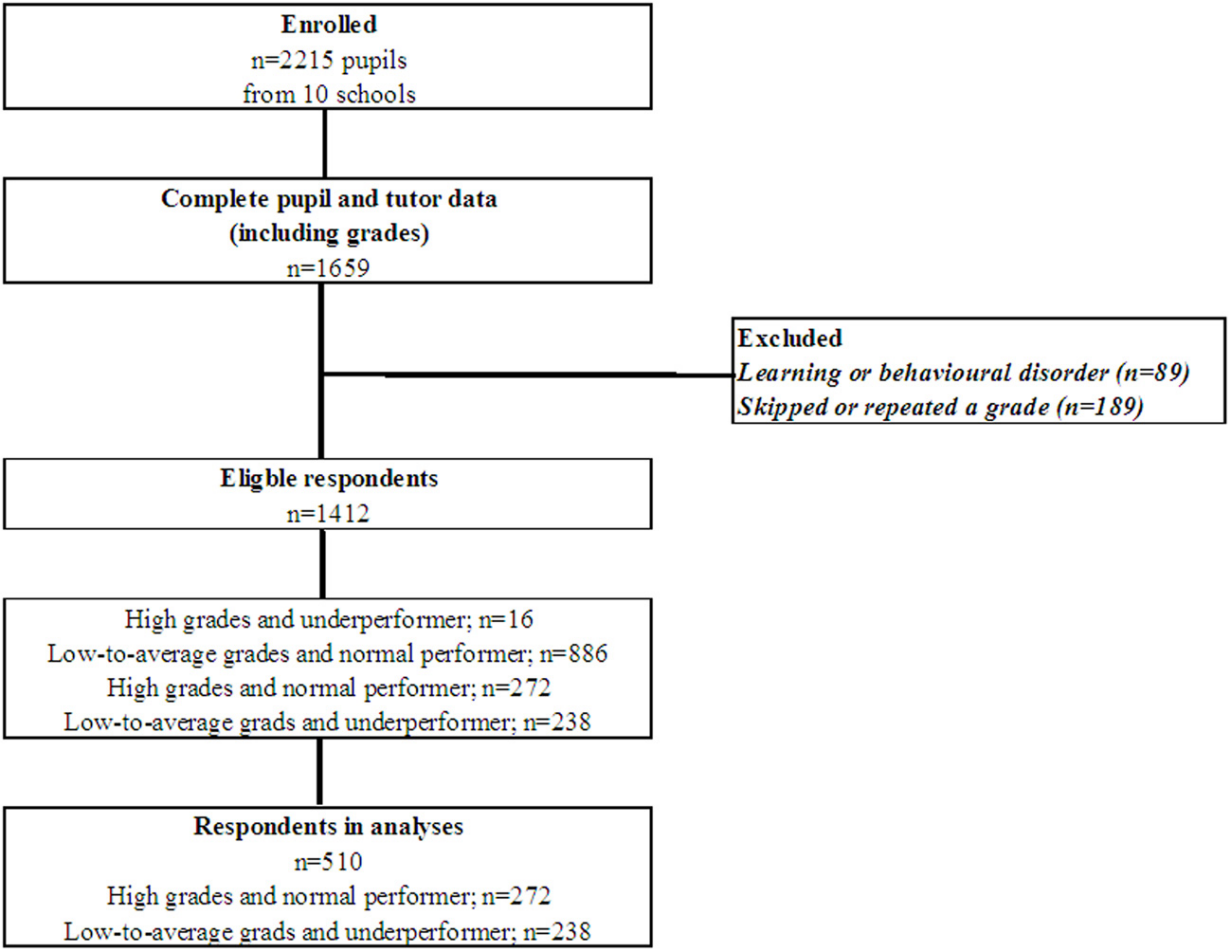
**Flow chart study population**.

In 556 pupils, the necessary data, i.e. self-reports, tutor reports, and grades, was incomplete or not available: (1) one school (*n* = 205 pupils) used a deviant grading policy, (2) for 310 pupils (in 14 complete classes), tutors failed to fill in the questionnaires and grades for the whole class, and (3) in 41 pupils (spread over 23 classes), tutor report and grades were missing for individual pupils. Thus, self-reports, tutor reports, and grades were available for 1659 pupils. Pupils with a learning disorders or behavioral disorder (*n* = 89) and pupils who skipped or repeated a grade after Kindergarten (*n* = 189) were excluded. We were interested in the factors that distinguished pupils with high grades who performed in line with their potential from pupils with low-to-average grades who did not perform in line with their potential. Therefore, pupils with high grades and who perceived themselves and were perceived by their tutors as underperformers were excluded (*n* = 16), as were pupils with low-to-average grades and whose performances were in accordance to their learning potential (according to themselves and to their tutors; *n* = 886). This resulted in 510 pupils in one or more analyses (see Figure 1).

### Procedure

Pupils were recruited from 10 schools situated in urban as well as rural regions in the Netherlands. Parents received a letter with information on the study and had to give active informed consent for their child's participation in the study. In the second semester (March/April 2011), participating pupils completed an online questionnaire in a computer room at their school. These sessions were supervised by a trained research assistant. Tutors also filled in online questionnaires about their pupils and provided pupils' grades.

### Emotional wellbeing

Pupils reported on their emotional wellbeing by filling in the online questionnaire. The scale measuring emotional wellbeing encompassed emotional symptoms (“I feel good and often I am happy,” “Quit often I am angry, sad, tense, or in a bad mood,” “Lately, I don't feel as good as I used to,” “If I worry I cannot stop thinking about it”), items on wellbeing (“I am satisfied with the things I do and the way I do these things,” “I like going to school”), and somatic complaints (“I often feel sick,” “I get a lot of headaches”). Items were scored on a 3-point scale ranging from 1 “not correct” to 3 “correct.” The coding of positively worded items was reversed. The values of the items were summed and divided by the number of endorsed items. A high score indicated less emotional wellbeing. In this study, the Cronbach's alpha for internal consistency was 0.72.

### Outcome: underachievement

Information on underachievement was collected with online questionnaires filled in by the pupils and by their tutors. Underachievement was defined as “a discrepancy between potential (or ability) and performance (or achievement)” (McCoach and Siegle, 2003), and was operationalized by combining several measures.

First, self-reported and tutor reported under performance in school were combined. Pupils rated the following statement: “I think I can do much better in school than I do at the moment.” Answering categories for this statement ranged from 1 “I totally disagree” to 6 “I totally agree.” For each pupil, tutors rated the following statement: “This pupil can do much better than his/her rates currently show. It's an underachiever” on a 5-point scale ranging from 1 “I totally disagree” to 5 “I totally agree.” When the pupil's own and the tutor ratings were consistent (i.e., when pupils scored “a sort of agree,” “agree” or “totally agree,” and tutors rated “agree” or “totally agree”), the pupil was classified as “an underperformer.” Any other combination of self- and tutor ratings were categorized as “no underperformer” or “normal performer.”

Second, in order to distinguish “high” and “low-to-average” achieving pupils, final grades for the subjects Dutch, English and mathematics for the first semester (September–December 2011) were used. Pupils were categorized as high achieving pupils, if their mean grade for Dutch, English and mathematics fell in the highest 20% of the distribution of grades within each class. Low-to-average achieving pupils had a mean grade for Dutch, English and mathematics in the lowest 80% of the distribution within each class. Pupils grades were categorized within each class to control for possible grading differences across classes or for the possible impact that interactions between pupils within a class may have on grades.

Finally, we combined the self- and tutor reported performance measure with achievement based on pupils' grades. Underperformers (i.e., in their own and in their tutor's perception) with low-to-average grades were classified as “underachievers,” whereas “normal performers” (i.e., who performed in their own and their tutor's view in accordance with their potential) with high grades were classified as “high achievers.”

### Covariates

Selection of probable confounders was based on earlier research. Information on the pupils' gender, age, ethnicity, educational level, self-reported neurocognitive functioning, i.e., attention problems, self-control and self-monitoring, and planning, cultural capital, attitude toward teachers, orientation on the future, time spent on sports was collected with online questionnaires filled in by the pupils. Gender and age were included as probable confounders, because of the marked sex differences in age of onset and prevalence in mental health disorders (Paus et al., 2008), in age at which puberty starts and in neurodevelopmental trajectories (Sisk and Foster, 2004). Educational level was included as a proxy of socio-economic status and consisted of six categories, i.e., “pre-vocational,” “pre-vocational/higher,” “higher,” “higher/pre-university,” “pre-university,” “pre-university with classical languages.” Some types of secondary schools in the Netherlands offer classes in the first grade with pupils of different levels. Therefore, some of the categories represent combinations of two educational levels. The Amsterdam Executive Function Inventory (AEFI) was used to assess three executive aspects of daily-life behavior, i.e., Attention, Self-Control and Self-Monitoring, and Planning and Initiative (Van Der Elst et al., 2012). The AEFI is a self-report questionnaire with adequate construct validity and reliability which has been described in detail elsewhere (Van Der Elst et al., 2012). In the current sample, Cronbach's alpha's for internal consistency were 0.79 for Attention, 0.60 for Self-Control and Self-Monitoring, and 0.61 for Planning and Initiative.

Cultural capital was included as a potential confounder because of its suggested relation with educational attainment and as a proxy for socio-economic status (De Graaf et al., 2000). Bourdieu's cultural capital hypothesis 1973, namely, posits that the effect of families' social background on educational attainment is partly explained by the increased access to cultural resources which aids children to master the curriculum in higher educational levels. Cultural capital was measured by the following items: “I enjoy reading a book,” “My parents like reading and culture,” “My parents used to read aloud to me,” and “With my parents, I sometimes visit a museum or theatre.”

On the basis of previous research (Midgley et al., 1989), we assumed an influence of attitude toward teachers on the relation between emotional wellbeing and achievement in school. To measure pupil's attitudes toward their teachers, pupils rated the following items: “My grades would be higher, if teachers would explain to me what I have to do,” “I think teachers should give me more freedom,” and “I think that teachers should show more interest in me and should give me more support.” The scale measuring “orientation on the future” comprised the following items: “I know what I want and how I can achieve my goal,” “I know very well what I want to become later in life,” “I haven't got a clue about what I want to do after this school.” All items were measured on a 3-point scale ranging from 1 “not correct” to 3 “correct.” Per scale items we calculated the average scores. In the current sample, Cronbach's alpha's for internal consistency were 0.59, 0.66, and 0.66 for cultural capital, satisfaction with teacher, and future orientation, respectively.

Finally, time spent on sports was included as a confounder because findings from previous studies suggest that physical activity is related to emotional or behavioral problems (Kantomaa et al., 2010), and because of its presumed impact on educational attainment (Pfeifer and Cornelißen, 2010). Time spent on sports was measured by asking pupils how much time per week they spent on sports activities. Answering categories were 1 “less than half an hour,” 2 “half an hour until an hour,” 3 “1–3 h,” 4 “3–6 h,” and 5 “more than 6 h.”

### Statistical analyses

We used chi-square tests and analysis of variance (ANOVA) for crude comparisons on selected variables between underachievers and high achievers.

Stepwise logistic regression analysis was used to determine the association between emotional wellbeing and underachievement. In the first model, the association of emotional wellbeing and underachievement was adjusted for background variables, gender, age, educational level, and ethnic background. In the second model, we added thee measures of self-reported neurocognitive functioning, i.e., Attention problems, Self-Control and Self-Monitoring, and Planning and Initiative. In the final model, we added cultural capital, attitudes toward teachers, satisfaction with the school and time spent on sports.

In addition, interaction terms of emotional wellbeing with gender and educational level were tested. Since all *p*-values exceeded 0.15, the interaction terms were not included in the models.

In our main analyses, underperformance was measured by combining self-reports and tutor-reports. Additionally, analyses were repeated for self-reports and tutor-reports separately.

### Response analyses

We investigated whether the excluded sample, i.e., normal performing pupils with low-to-average grades (*n* = 886) differed from the two groups comprising the included sample, i.e., the high achievers and underachievers (see Table 1: comparison a and b). In comparison to the high achievers, excluded pupils had more attention problems, more problems with Self-Control and Self-Monitoring, and had a more negative attitude toward their teachers, but compared to the underachievers, the excluded pupils felt better, had less attention problems, less problems with Self-Control and Self-Monitoring, and had a more positive attitude toward their teacher (see Table 1).

**Table 1:** **Sample characteristics and univariate comparisons of non-response and response groups**.

	**Non-response**	**Response**	
	**Normal performer with low-to-average grades *n* = 886**	**High achiever^#^*n* = 272**	**Underachiever^§^*n* = 238**
Emotional wellbeing score; mean (*SD*)	1.62 (0.40)^a^^,^^b^	1.54 (0.34)^a^^,^^c^	1.77 (0.41)^b^^,^^c^
Female, %	47%^a^^,^^b^	57%^a^^,^^c^	34%^b^^,^^c^
Age, years; mean (*SD*)	12.5 (0.6)^b^	12.4 (0.6)^c^	12.7 (0.6)^b^^,^^c^
Secondary educational level, %			
Pre-vocational	9%	9%	7%
Pre-vocational/higher general	14%	15%	20%
Higher general	8%	9%	5%
Higher general/pre-university	28%	27%	37%
Pre-university	7%	8%	10%
Pre-university with classical	34%	32%	21%
languages	X^2^_b_	X^2^_c_	X^2^_b,c_
Ethnic background, %			
Dutch	84%	83%	80%
Non-Dutch, western	10%	10%	13%
Non-Dutch, non-western	6%	7%	7%
Attention problems; mean (*SD*)	1.93 (0.62)^a^^,^^b^	1.73 (0.57)^a^^,^^c^	2.23 (0.59)^b^^,^^c^
Self-control and self-monitoring; mean (*SD*)	1.76 (0.41)^a^^,^^b^	1.60 (0.35)^a^^,^^c^	1.94 (0.41)^b^^,^^c^
Planning and initiative; mean (*SD*)	2.30 (0.39)	2.34 (0.40)^c^	2.26 (0.38)^c^
Cultural capital score; mean (*SD*)	2.26 (0.50)^b^	2.28 (0.45)^c^	2.16 (0.50)^b^^,^^c^
Attitude toward teacher score; mean (*SD*)	1.81 (0.57)^a^^,^^b^	1.66 (0.50)^a^^,^^c^	2.00 (0.55)^b^^,^^c^
Orientation on the future score; mean (*SD*)	2.11 (0.56)	2.07 (0.58)^c^	2.16 (0.57)^c^
Time spent on sports activities, %			
<1 h per week	13%	16%	11%
1–<3 h per week	30%	26%	27%
3–<6 h per week	36%	39%	38%
6 h or more per week	22%	19%	24%

# High achiever: normal performer, i.e., not an underperformer, with high grades.

§ underachiever: underperformer, i.e., performing worse than expected based on his/her capabilities.

a significant difference between normal performing pupils with low-to-average grades and those with high grades.

b significant difference between pupils with low-to-average grades normal performers and those who are underperformers.

c significant difference between the two included samples, i.e., high achievers vs. underachievers.

a, b, c: p < 0.01.

M, mean; SD, standard deviation.

Chi-square tests were used for categorical variables, ANOVA was used for continues variables.

## Results

In Table 1, the univariate comparisons of the response groups are shown. Underachieving pupils felt less well, were more often boys, had more attention problems, had more problems with Self-Control and Self-Monitoring, were provided with less cultural capital and had a more negative attitude toward their teachers than the high achieving pupils [emotional wellbeing *F*_(1, 510)_ = 43.9; gender *X*^2^ = 27.9; attention problems *F*_(1, 510)_ = 93.9; Self-Control and Self-Monitoring *F*_(1, 510)_ = 103.3; attitude teacher *F*_(1, 510)_ = 54.1; all *p*-values < 0.001].

In Table 2, associations between emotional wellbeing and underachievement are presented for the three successive models. First, higher scores on emotional wellbeing (i.e., feeling less well) were associated with an increased risk of underachievement (OR 5.15, 95% CI 3.06–8.68) independent of several background variables. Furthermore, male and older age were associated with an increased risk of underachievement (see Table 2). Second, by adding measures of neurocognitive functioning to the model, the association between emotional wellbeing and underachievement attenuated considerably (OR 2.22, 95% CI 1.23–3.99). Attention problems and problems with Self-Control and Self-Monitoring were associated with an increased risk of underachievement (attention OR: 2.22, 95% CI 1.44–3.43; Self-Control and Self-Monitoring OR: 3.57, 95% CI 1.85–6.89). Third, in the final model, the last group of confounders was added. This further attenuated the association between emotional wellbeing and underachievement (OR 1.99, 95% CI 1.08–3.66). Of these confounders, a more negative attitude toward teachers and being oriented at the future was associated with an increased risk of underachievement (attitude toward teacher OR: 1.79, 95% CI 1.17–2.75; orientation on the future OR: 1.63, 95% CI 1.11–2.41).

**Table 2:** **Stepwise logistic regression analyses showing the association between emotional wellbeing and underachievement including the changes in this relation by adding confounding variables**.

	**Risk of underachievement compared to high achievers^a^**								
	**Adjusted for background variables**			**Additionally adjusted for neurocognitive functioning**			**Additionally adjusted for other confounders**		
	**OR**	**95% CI**	***p***	**OR**	**95% CI**	***p***	**OR**	**95% CI**	***p***
Emotional wellbeing	5.15	(3.06–8.68)	<0.001	2.22	(1.23–3.99)	0.008	1.99	(1.08–3.66)	0.028
Gender									
Female	1	Reference	<0.001	1	Reference	<0.001	1	Reference	<0.001
Male	2.95	(1.99–4.38)		2.81	(1.84–4.30)		2.58	(1.65–4.04)	
Age	1.72	(1.22–2.43)	0.002	1.69	(1.17–2.44)	0.005	1.66	(1.13–2.42)	0.009
Secondary educational level	0.92	(0.82–1.03)	0.15	0.94	(0.83–1.07)	0.38	0.98	(0.85–1.12)	0.75
Ethnic background									
Dutch	1	Reference		1	Reference		1	Reference	
Non-Dutch, western	1.26	(0.68–2.31)	0.46	1.40	(0.72–2.73)	0.32	1.44	(0.72–2.87)	0.32
Non-Dutch, non-western	0.91	(0.43–1.96)	0.82	1.09	(0.48–2.49)	0.83	1.07	(0.47–2.47)	0.87
Attention problems		–		2.22	(1.44–3.43)	<0.001	2.12	(1.36–3.29)	0.001
Self-control and self-monitoring		–		3.57	(1.85–6.89)	<0.001	3.09	(1.57–6.09)	0.001
Planning		–		0.95	(0.53–1.70)	0.86	0.68	(0.35-1.30)	0.24
Cultural capital		–			–		0.94	(0.56–1.56)	0.80
Attitude toward teacher		–			–		1.79	(1.17–2.75)	0.007
Orientation on the future		–			–		1.63	(1.11–2.41)	0.014
Time spent on sports activities									
<1 h per week		–			–		0.72	(0.34–1.55)	0.40
1–<3 h per week		–			–		1.13	(0.61–2.09)	0.70
3–<6 h per week		–			–		1.02	(0.57–1.80)	0.96
6 or more h per week		–			–		1	Reference	

OR, odds ratio; CI, confidence interval.

n = 510 in one or more analyses.

a A pupil was classified as an underachiever if he/she and the tutor rated the pupil as performing lower than the pupils potential in combination with low to average grades. A pupil was classified as a high achiever if he/she was not rated as performing lower than he or she could and if the pupil had high grades.

In Table 3 results are shown for the associations between emotional wellbeing and the risk of underachievement calculated in two different ways. When using only tutor reports to measure underperformance, results were highly similar to those of the combined measure of pupil's and tutor reports. When only the pupil's reported underperformance was used, results were still very similar, yet the final model failed to reach significance.

**Table 3:** **Logistic regression analyses showing the association between emotional wellbeing and underachievement reported by tutors and reported by pupils**.

	**Adjusted for background variables^#^**			**Additionally adjusted for neurocognitive functioning^$^**			Additionally adjusted for other confounders^*^		
	**OR**	**95% CI**	***p***	**OR**	**95% CI**	***p***	**OR**	**95% CI**	***p***
**RISK OF UNDERACHIEVEMENT (REPORTED BY TUTORS) COMPARED TO HIGH ACHIEVERS^a^**									
Emotional wellbeing	4.45	(2.70–7.31)	<0.001	2.00	(1.14–3.52)	0.016	1.79	(1.00–3.20)	0.048
**RISK OF UNDERACHIEVEMENT (REPORTED BY PUPILS) COMPARED TO HIGH ACHIEVERS^a^**									
Emotional wellbeing	5.69	(3.31–9.78)	<0.001	1.98	(1.09–3.62)	0.026	1.71	(0.92–3.16)	0.089

OR, odds ratio; CI, confidence interval.

n = 541 for tutor report; n = 918 for pupils report.

a A pupil was classified as an underachiever if he/she and the tutor rated the pupil as performing lower than the pupils potential in combination with low to average grades. A pupil was classified as a high achiever if he/she was not rated as performing lower than he or she could and if the pupil had high grades.

# Models adjusted for gender, age at assessment, secondary educational level, ethnic background.

$ Models # additionally adjusted for self-reported neurocognitive functioning, i.e., attention problems, self-control and self-monitoring and planning and initiative.

* Models $ additionally adjusted for cultural capital, attitude toward teacher, orientation on the future, time spent on sports.

## Discussion

In this large scale study of early adolescent pupils, we found that emotional wellbeing was substantially related to the risk of underachievement in school. This relation was partly explained by self-reported neurocognitive functioning. Adding other covariates, such as attitude toward teachers and orientation on the future further weakened the association between emotional wellbeing and underachievement in school, but the association remained significant.

Fluctuations in mood are very common and may originate from exposure to potentially stressful events that are part of everyday life, i.e., daily hassles. EF play an important role in the extent to which common fluctuations in mood impact on daily life functioning such as achievements in school. For example, inhibiting behavioral impulses, regulating stress and frustrations and problem solving are EF which increase resilience to emotional problems (Riggs et al., 2006). We observed a relation between mildly reduced emotional wellbeing and underachievement in school, which was only partly explained by self-reported neurocognitive functioning. Our measure of neurocognitive functioning consisted of three important components of executive aspects of daily life behavior, i.e., Attention, Self-Control and Self-Monitoring, and Planning and Initiative (Van Der Elst et al., 2012). It is possible that these self-report measures of executive aspects of daily life behavior do not cover the full range of EF. Other aspects of EF may additionally explain the association between emotional wellbeing and underachievement in school.

Transferring from one school to another is considered a major life event (Felner et al., 1981, 1982). Enrolling in secondary education probably has a larger impact than just changing to a new school. As all pupils enter secondary education simultaneously and attempt to adapt to the new setting at the same time, the whole school setting generally is in a state of flux (Felner et al., 1982). This less stable and unpredictable environment demands many adaptations and high levels of flexibility, and may temporary or not increase levels of not feeling very well and may cause pupils to perform less well than is to be expected based on their potential. Although the transition to secondary education may have contributed to the observed relation between emotional wellbeing and underachievement, again the coping and its relatedness to neurocognitive functioning determines the extent to which such a life event has consequences for emotional wellbeing and underachievement. Future research conducted in a sample of pupils further in their secondary education can shed light on the extent to which our findings are mainly due to the transition to high school that pupils have undergone.

Entering secondary education is a large impact on young adolescents' lives, but, what is more, in the Netherlands, the transition to secondary education co-occurs with the average age at which puberty starts (Mul et al., 2001). The developmental stage from childhood to adulthood is characterized by dramatic alterations in physical appearance and behavior. These changes are accompanied by rapid developmental changes in the brain and how the brain functions (Blakemore et al., 2010; Crone and Dahl, 2012). How adolescents go through this developmental stage is essential for their future. Reduced emotional wellbeing which is quite common can be a precursor of later mental health problems (Kessler et al., 2005). Findings from a series of studies suggest adolescent-associated increases in sensitivity to stressors and affect responsivity (Crone and Dahl, 2012). These may aid most adolescents in coping with increasingly diverse environmental circumstances, but may in some high-risk adolescents enhance individual predisposition to mental health problems by dysregulating stress responses (Dahl and Gunnar, 2009; Spear, 2009). We investigated a typical developing population of pupils in early adolescence and found a substantial relation between mildly reduced emotional wellbeing and educational underachievement. Through educational attainment and via more serious mental health problems, emotional wellbeing is a crucial factor in early adolescence, as both factors are strongly related to later health, socio economic status, social relations (Hartog and Oosterbeek, 1998; Mirowsky and Ross, 2003).

Most research on the effect of mental health problems in adolescents on educational attainment focuses on the high end of the distribution of problems, i.e. on mental health disorders (for example, Breslau et al., 2008; Lee et al., 2009; Humensky et al., 2010). The results of these studies suggest that the effect of externalizing disorders on educational attainment was most profound whereas for internalizing problems the effect on educational attainment was less clear. The studies that investigated the effect of less severe problems instead of disorders are also focused on externalizing problems—effects of internalizing problems are relatively understudied in this field- or on childhood behavioral and emotional problems (Rutter, 1974; Hinshaw, 1992; McLeod and Kaiser, 2004; McLeod and Fettes, 2007). Thus, the current study is unique in that we observed a relation between mild deviances from emotional wellbeing and underachievement in school. In addition, this relation was independent of self-reported neurocognitive functioning, attitude toward teachers, and future orientation.

In this study, we found that the relation between emotional wellbeing and underachievement was partly explained by aspects of executive functioning. These findings are, in part, in line with the model of self-regulated learning. According to Zimmerman (2008), self-regulated learning may be defined as self-generated thoughts, actions, and feelings which are intended to achieve specific educational goals under conditions of individual choice and control. Self-regulatory behaviors include goal setting, planning, decision making, using cognitive strategies, self-evaluation, self-monitoring, eliminating competing demands and systematically adapting learning efforts on the basis of learning outcomes (Zimmerman, 2008). Although it is imaginable that decreased emotional wellbeing obstructs the self-regulated learning strategies necessary to achieve academic goals, comparing our findings with the self-regulated learning model is difficult, as our measures only tap some of the constructs included in this model.

This study has several strong features. First, data from a large general population sample was used. This sample consisted of early adolescent pupils who just made the transition to secondary education and who were relatively homogeneous in age. Second, we collected data from two different informants, i.e., the pupils and their tutors. This enabled us to calculate a combined measure of underachievement based on the pupil and tutor reports and calculate separate measures for each informant. Using the same informant to measure determinants and outcomes may result in reporter bias. Finally, in this study we took a multidisciplinary perspective by investigating the extent to which the relation between emotional wellbeing in young adolescents and underachievement in high school was explained by self-reported neurocognitive functioning.

Several methodological limitations need to be discussed as well. First, although we took account of many different confounders, we cannot rule out that residual confounding affected our findings (Rothman et al., 2008). For example, social economic status was not included in our study. However, in our analyses we did correct for educational level of the pupils which is strongly related to parental educational level and thus, can be regarded as an indicator of social economic status. Second, there may be better indicators to measure learning potential than school grades. Third, the emotional well-being questionnaire has good internal consistency, however, it requires further psychometric validation through future research. Fourth, some selection bias may have occurred due to incomplete or missing tutor reports or grades. A tutor in the Netherlands is, besides a teacher, appointed to coach all pupils in a class. Thus, he/she sees pupils during classes and personal meetings on a regular basis. This study was conducted halfway the second semester of the school year. As a consequence, tutor and pupils knew each other for approximately 8 months, which is almost a whole school year. We collected no information on the quality of the relationship between pupils and tutors. It may be possible that this relationship affected the selection process. However, this is unlikely, as the majority of dropout due to missing tutor reports or grades occurred in classes as a whole and not for individual pupils. Fifth, we cannot completely rule out that some results are less accurate as several covariates such as cultural capital, self-control and self-monitoring, and planning and initiative had low internal consistencies (0.59–0.61). Finally, because of our cross-sectional design, we cannot make any inferences on causality or directionality. For example, it is feasible that reduced emotional wellbeing increases the risk of underachievement, but it is equally possible that underachievement increases the risk of problems with emotional wellbeing. Further research is necessary to shed more light on the underlying mechanisms.

In conclusion, in improving school achievement or reducing the number of students who perform below their potential, emotional wellbeing seems to play an essential role. The observed findings suggest that the risk of underachievement may be reduced by improving some neurocognitive functions, such as attention problems and problems with self-control and self-monitoring. Also, the relation with a teacher and attitude toward a teacher may motivate pupils to perform in school in accordance with their potential. Finally, screening for emotional wellbeing and improving feelings of worthlessness in young adolescents is probably worthwhile.

### Conflict of interest statement

The authors declare that the research was conducted in the absence of any commercial or financial relationships that could be construed as a potential conflict of interest.
